# Orthopedic Considerations in the Pedestrian versus Motor Vehicle Accident Polytrauma Patient

**DOI:** 10.1155/2012/149847

**Published:** 2012-06-17

**Authors:** Jason Samona, Robert Colen

**Affiliations:** ^1^College of Osteopathic Medicine, Michigan State University, East Lansing, MI 48824, USA; ^2^Botsford General Hospital, Farmington Hills, MI 48336, USA

## Abstract

Pedestrian versus motor vehicle accidents (PVMVAs) are a common cause of morbidity and mortality around the globe. Past models of PVMVAs assume lower-extremity vehicle contact as the initiating event, with a subsequent predicted injury sequence consisting of a lower extremity injury followed by injury to the body, head, and upper extremities. The term “fatal triad” was first coined by Farley, which described concomitant injuries to the skull, pelvis, and extremity fractures. Over the years, this once well-accepted model of injury has been under scrutiny by numerous orthopedic researchers, and it has lost credibility. This case presentation glaring reveals that the patient incurred which is referred to as the “fatal triad”, in contrast to the commonly circulated thoughts of biodynamic mechanisms of PVMVA fractures. More research in this arena is warranted. This lack of information contributes to the morbidity and mortality associated with such devastating injuries. The overlying theme displayed in the data analyzed in this paper demonstrates the vital importance of the orthopedic surgeon in the management of the PVMVA patient. No matter the particular mechanism of injury, occurrence, or agreed-upon treatment protocol, the role of the orthopedic physician is instrumental to the wellbeing of the PVMVA trauma patient.

## 1. Introduction

Pedestrian versus motor vehicle accidents (PVMVAs) are a common cause of morbidity and mortality around the globe. In 2007, there was 70,000 pedestrians injured and 4,654 killed in PVMVAs in the United States alone [1]. The occurrence is so common; it is speculated that 1 pedestrian is killed by a motor vehicle every 112 minutes and injured every 8 minutes in the US [2]. From 1993 to 2003, a multicenter study in the US including 5338 PVMVA victims revealed about 20% of patients to be less than 14 years, 64% were 15–55 years, 7% were 56–65 years, and 9% greater than 65 years of age [2]. A significant number of trauma admissions and deaths in urban areas are accredited to PVMVAs. These are astonishing numbers, but even more surprising is that PVMVAs are an even larger problem in the developing world, where mortality rates reach as high as 40 per 100,000 persons per year. This is partially due to the rapid motorization and the inattention of policy makers to address these issues in many developing nations [3]. Overall death from PVMVA is 7.7%, but this increases with age, with an average of 3.2% among the pediatric population versus 25.1% among individuals greater than 65 years of age. The overall theme is that, the older the patient, the greater the risk of serious injury and death [2].

Pedestrian versus motor vehicle accidents are a very common cause of trauma admission and subsequent orthopedic consultation. Patients can have multiple injuries and be difficult to evaluate initially upon presentation to the local trauma center. Pain medication administration and immobility leading to lack of symptoms and associated lab abnormalities, along with reduced access to imaging in the unstable patient, further complicate the diagnosis of orthopedic injuries in this patient population. Due to these factors inhibiting proper treatment of the orthopedic trauma patient, clinical researchers, and policy makers have instituted “biodynamic constructions” [4] and epidemiological reviews to identify predictors of orthopedic injury following PVMVAs. The models were established to better anticipate associated injuries with PVMAs and therefore improve the level of care to these patients. These biodynamic constructions describe relationships which exist between physical objects in an event as they relate to the patient and the nature of the impact. Past models of PVMVAs assume lower-extremity vehicle contact as the initiating event, with a subsequent predicted injury sequence consisting of a lower extremity injury followed by injury to the body, head, and upper extremities [4]. The term “fatal triad” was first coined by Farley [5], which described concomitant injuries to the skull, pelvis, and extremity fractures, with an associated mortality of 25% [5]. Waddell and Drucker later redefined the components of a triad through his own research. He claimed injuries to the head, pelvis/hip, and knee region better described the “triad,” and he reported one fatality in a review of 10 cases. Waddell was as bold as to proclaim that this triad was present in all serious PMVAs [6]. Over the years this triad has been under scrutiny by numerous orthopedic researchers [4, 7, 8]. Once a well-accepted model of injury, it has lost credibility due to decreased confidence on the reliance of theory. Landy et al., in a review of 336 cases of PVMVA spanning 2 years at a level 1 trauma center, claimed that many PVMVA cases do not resemble the currently proposed biodynamic models which support the fatal dyad hypothesis [4]. Brainard et al. in a retrospective review spanning 2 years and 115 PVMVAs revealed the incidence of Waddell's triad in his study to be only 9%. He claims there was no statistical correlation between head trauma and the presence of a knee or pelvis/femur/tibia fracture. Brainard and other researchers have also noted a high occurrence of femur fractures with associated pelvic fractures in PVMVAs patients, as well as lower-extremity and ipsilateral upper-extremity injury, referred to as the “ipsilateral dyad” [4, 7]. The results demonstrate that many PVMVAs likely do not involve lower extremity contact as the initiating event, and previous models of injury should be disregarded.

PVMVA studies reveal the rates of occurrence of particular injuries, such as craniocerebral injury at 57.1%, thoracic injury at 31.2%, spinal injury at 5.1%, extremity injury at 35.1%, and overall mortality at 11.7%. The most common area of “serious/life threatening injury” was found to be the head, which is in stark contrast to the chest (2.7%), abdomen (2.1%), and extremities (1.2%). Head injuries increase significantly with age. They are approximately 3 times more likely in patients greater than 65 years of age as compared to individuals 14 years of age and younger. Subdural and subarachnoid hemorrhages especially were noted to increase markedly with increased age [2]. Markogiannakis has analyzed 77 pedestrians PVMVA cases, to reveal that craniocerebral injuries were the leading cause of death, as demonstrated by numerous other studies as well. Of the deceased pedestrians in this particular study, 66.7% died due to head trauma, 22.2% as a result of hemorrhagic shock (attributable to a concomitant thoracic, abdominal, and/or pelvic injuries) and 11.1% from septic shock [9]. These findings are in concordance with other studies [10].

The incidence of spinal injuries is only 5.1% in PVMVAs, with no difference of occurrence at cervical, thoracic, or lumbar spinal regions. Risk of spinal injury was 21 times greater in patients greater than 65 years of age at 8.5%, as compared to the pediatric population at 4%. The occurrence increases dramatically with age, partially due to factors including osteoarthritis and osteoporosis contributing to susceptibility to injury.

Evaluation of abdominal trauma revealed liver injuries at 2.4%, splenic injuries at 1.7%, renal injuries at 8%, and gastrointestinal injuries at 4.1% of PVMVAs. With regards to abdominal trauma, no significant difference exists across age groups. Pelvic injuries occur in 12.8% of patients and are 3.5 times more likely to be found in patients greater than 65 years of age, versus children under 14 (6.3% occurrence). The pelvic trauma patients typically complain of severe back, abdominal, or suprapubic pain. Although in the unconscious polytrauma patient, these complaints are not commonly expressed upon initial presentation. Of the lower extremities, tibial fractures are the most common injury, accounting for 25.9% [2].

Management of patients with polytrauma is complicated by the presence of pelvic fractures and the associated visceral, vascular, and/or neurological injury. When the pelvic fracture is discrete, management follows widely accepted treatment protocols. Overall, 40% of patients with pelvic fractures also have abdominal injuries [11]. The force required to fracture the pelvic ring is big, making associated injury common. In scenarios of pelvic ring disruption, associated injuries occur in more than 90% of patients. This is commonly seen in the PVMVA victim who incurs severe trauma (such as in this patient), in which visceral, vascular, and/or neurological injury is associated. In these cases the treatment protocol is not as widely accepted due to difficulty in evaluation of the patient, varying degrees of severity of multiple injuries, the ability to gauge the life-threatening status of numerous injuries, institutional capabilities, health care provider experience, and so forth. These trauma patients with intra-abdominal blood and pelvic fractures present a diagnostic and management challenge for which no clear consensus on the preferred approach exists [12, 13]. Head injury is associated in 27% of such patients and thoracic injury in 26%, and in regards to anteroposterior pelvic injury an 800% increase in aortic rupture has been documented [11]. Lumbosacral plexus and nerve root neurologic injuries may be present but may not be apparent in the unconscious patient for obvious reasons. GI and GU injury is not uncommon in the patient suffering from pelvic ring disruption. Bladder injury occurs at the high incidence of 20% and urethral injury at 10% (more common in males). Bowel injuries are not uncommon either in the pelvic ring fracture patients. Razor sharp osseous fragments leading to perforations in the rectum or anus owe to technically “open injuries” and therefore should be treated as such. Infrequently, severe cases of entrapment of bowel in the actual fracture site with subsequent gastrointestinal obstruction may occur [14].

## 2. Case Report

A 61-year-old African American female was found unconscious on the road after a suspected pedestrian versus motor vehicle accident. The patient was supposedly walking alongside a busy 4 lane road in the early evening time, when she was struck by a driver. EMS was notified by pedestrians, and she was found in the street unconscious at the time they arrived. There were no witnesses to the accident which came forward to aid police or medical professionals at the scene, and the vehicle involved in the incident fled. Specific details regarding the injury itself are unknown. The patient was intubated by EMS in the field and was brought to the local trauma center.

At the time of presentation to the ED, the blood pressure was 64/50 (posttransfusion and placement of pelvic binder; BP reached 150/110), heart rate of 119, and respiratory rate of 22. The abdomen was soft, nontender and nondistended. No signs of rigidity or acute abdominal trauma were seen at this time. There was an open left lower leg fracture medially with an approximately 15 × 8 cm open wound with “venous oozing.” GCS of 7 quickly deteriorated to GCS of 3. Rectal exam revealed “no” tone. There was no gross blood noted per rectum, but the patient is guaiac positive. There was no vaginal blood or blood in the urethral meatus; however, later the patient was found to have gross bright red blood draining from the catheter.

Hemodynamic instability upon arrival was apparent, and resuscitation through advanced life support (ATLS) protocol was quickly initiated shortly after arrival to the ED. IV fluids and trauma blood were given; 18 units of packed red blood cells, 11 units of FFP, and 1 unit of trauma platelets were been given to this point.

The patient was transported to angiogram for angiography of the vertebral artery and embolization due to pelvic bleeding. Images showed large amounts of extravasation of contrast in the right lower quadrant corresponding to areas identified on a recent CT. The right internal iliac artery was then selectively catheterized via interventional radiology procedures. Spot images were obtained after embolization, demonstrating a stop to the bleeding branch. An injection of contrast showed complete resolution of the bleed in the pelvis. The bleed probably represented hemorrhage from the inferior gluteal artery. A very tiny blush of contrast was demonstrated in the midpelvic region. It was elected not to embolize this area, as it would have required proximal embolization of the entire right internal iliac system. This action would have diminished the patient's opportunity for healing after pelvic reconstructive surgery. Narrowing of the right distal external iliac artery and common femoral artery was seen on fluoroscopy, thought to be due to extrinsic compression from a hematoma. The patient tolerated the procedures and remained hemodynamically stable throughout the process. She was transferred to the intensive care unit in what was considered hemodynamically stable condition. In summary, the patient had a hemorrhage in the right lower quadrant secondary to pelvic trauma which was successfully embolized.

**Figure 1:**
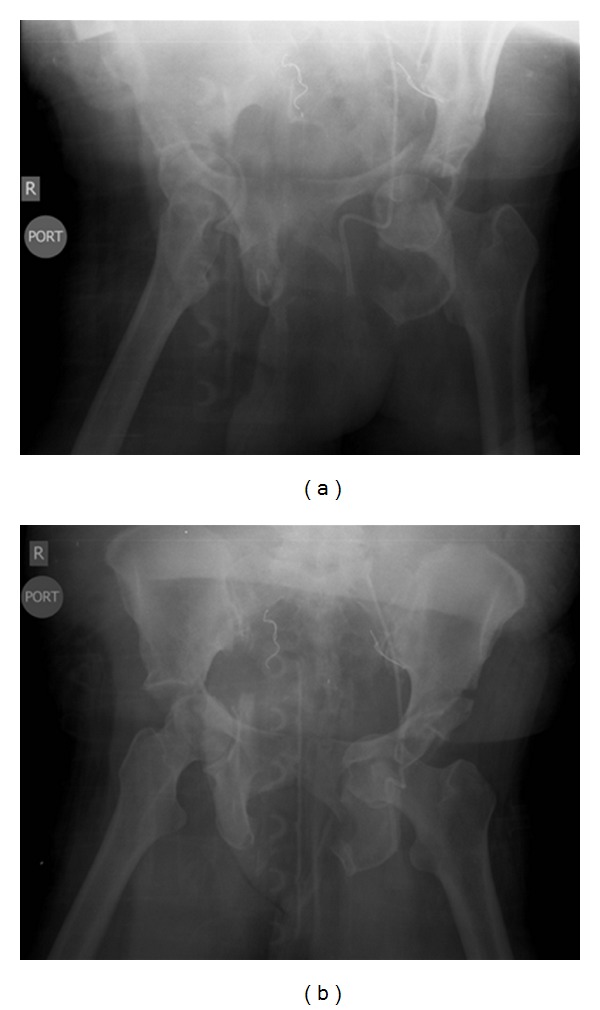
(a) Anterior-posterior radiographic image of pelvis—courtesy of the Botsford General Hospital Radiology Department. (b) Anterior-posterior radiographic image of pelvis—courtesy of the Botsford General Hospital Radiology Department.

**Figure 2:**
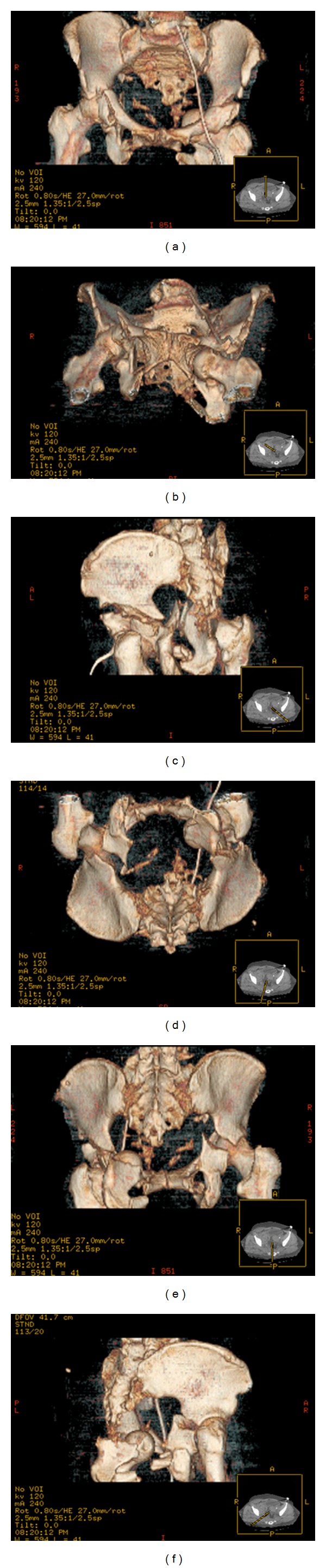
(a) 3D CT reconstruction of pelvis—courtesy of the Botsford General Hospital Radiology Department (anterior-posterior view). (b) 3D CT reconstruction of pelvis—courtesy of the Botsford General Hospital Radiology Department (anterior-inferior view). (c) 3D CT reconstruction of pelvis—courtesy of the Botsford General Hospital Radiology Department (lateral: left to right view). (d) 3D CT reconstruction of pelvis—courtesy of the Botsford General Hospital Radiology Department (inferior view). (e) 3D CT reconstruction of pelvis—courtesy of the Botsford General Hospital Radiology Department (posterior-anterior view). (f) 3D CT reconstruction of pelvis—courtesy of the Botsford General Hospital Radiology Department (lateral: right to left view).

Multiple images were taken of the patient in order to direct appropriate surgical and medical management (Figures 1 and 2). A CT brain without IV contrast revealed a subdural hematoma, fracture of the left zygomatic arch and left lateral orbit, but no depressed skull fracture. A single frontal view chest X-ray revealed no acute disease/process. Shoulder X-rays revealed no evidence of fracture or dislocation. Knee X-rays revealed no evidence of fracture or dislocation. Tibia/fibula X-rays demonstrated comminuted fractures seen to involve the distal shaft of the left tibia. At least 3 fracture sites were noted in the fibular shaft which were also comminuted, involving the mid- and distal portions. Normal alignment remained at the ankle joint and knee joint as noted by imaging. Soft tissue edema was present at the left lower extremity. The open fracture of the distal lower leg had a large opening medially, which was approximately 15 × 8 cm in length. Bony fragments were present. All compartments were soft upon physical exam. A nonenhanced CT of the cervical spine and pelvis with additional 3D-volume rendered reformatted images was obtained. This displayed a minimally displaced fracture of the right C7 transverse process, traversing the right vertebral artery foramen. No traumatic subluxation was seen in the spine. A CT thorax with IV contrast via trauma protocol was performed, revealing no evidence of pneumothorax, pleural effusion, or pulmonary contusion, normal-appearing mediastinum and great vessels with bovine type arch, and no evidence of osseous trauma.

A pelvic X-ray showed multiple pelvic fractures. CT abdomen and pelvis with IV contrast via dedicated trauma protocol was also performed. This revealed bilateral transverse process fractures of L5 and left transverse process fractures of L3-4. Comminuted pelvic fractures were evident. Bilateral comminuted acetabular fractures with protrusion of the left femoral head into the pelvis were seen. Bilateral pubic rami fractures were noted. Disruption of the left sacroiliac joint, anteriorly and posteriorly, was seen. A complex-free fluid was seen adjacent to the urinary bladder just below the pelvic brim on the right, likely representing blood and a pelvic hematoma. Via the Letournel classification of acetabular fractures, there was a right-sided T-type acetabular fracture, with communication at the anterior acetabular wall, as well as a left-sided T-type fracture with communication at the anterior and posterior acetabular walls. The left femoral head and floor of the acetabulum were medially displaced into the true pelvis. It was also apparent that there was a fracture at the junction of the left inferior pubic rami and left ischial tuberosity. Via the Young and Burgess Classification of Pelvic Ring Fractures, it could be noted that there was a *p*elvic *l*ateral *c*ompression *t*ype II *i*njury on the left, *p*elvic *l*ateral *c*ompression *t*ype I *i*njury on the right, as well as a *p*elvic *l*ateral *c*ompression *t*ype III *i*njury. A broad term to explain the pelvic trauma could be the classification designation as a “*c*ombined *m*echanical” injury to the pelvis.

Despite the numerous life saving measures enacted in the treatment of this patient, she subsequently passed away from her injuries. She went into a hypotensive crisis and cardiac arrest. The supposed location of hemorrhage leading to hypotension was thought to be secondary to a spinal injury to the transverse process of C7, causing dissection of the vertebral artery.

## 3. Discussion

The findings from the patient support the biodynamic profile explaining the nature of PVMVA trauma proposed by Farley and later revised by Waddell. This theory was once well accepted in the orthopedic community but has been scrutinized by numerous academic orthopedic sources. The patient sustained an open fracture to the tibia/fibula and substantial pelvic trauma, as well as extensive head injuries in the form of intracranial hemorrhage and orbital and periorbital fractures. The pattern of injury in this patient is in full support of the triad described by these early pioneers in trauma research. Ironically, despite the high degree of trauma incurred, the patient did not display a femur fractures with the associated pelvic fracture or the “ipsilateral dyad,” which modern medical literature has began to accept as common place among PVMVAs. The caveat to the patient in this case report is the severity of injuries. While PVMVA is not necessarily a rare occurrence, the severity of the injuries this patient incurred as a result of the incident is not particularly common. Thus this patient is unique as compared to the majority of the PVMVA cases analyzed in the current medical literature. The authors of this paper propose that the triad of findings as described by Farley and Waddell may possibly pertain more so to extensive PVMVA victims. The current medical literature lacks substantial and definitive research in this arena. It is evident that the currently circulated biodynamic models of PVMVA injury need to be reviewed and revised.

Causes of injury and death in PVMVA are of great concern to the health care provider. The orthopedic surgeon should know his role in the chain of healthcare professionals and therefore be ready to give pertinent advice and definitive treatment when the time calls. By analyzing the types of injuries typically incurred by PVMVA and the mortality rates of the associated injuries, treatment protocols may be formed to improve patient care. The patient in question was found to have a head injury (the most common in PVMVAs), but the most serious injuries were those at the pelvis and spine. Only 5.1% of PVMVA patients are found to have spinal injury, but this patient had multiple injuries to her spine which ultimately were the cause of her death due to associated vascular complications. The combined vascular insults, incurred in the pelvis and spine with subsequent blood loss, sent the patient into a hemorrhagic hypovolemic shock, which ultimately led to the patient's death. This is in difference to the nationally accepted statistics, in which craniocerebral injuries are the largest proportion of injuries incurred and by far the most common cause of death in the PVMVA patient. The patient was an elderly woman, had documented multiple craniocerebral injuries, including intracranial, orbital, and periorbital trauma, and thus would lead one to believe that the highest risk of death lies in the effects caused to the head. Where previous multistudy centers have shown 66.7% of PVMVA deaths to be due to head trauma as compared to 22.2% resulting from hemorrhagic shock, the extent of the orthopedic injuries should be taken into account when evaluating the true risk of death per injury. However, one must keep in mind that the studies which derived this information do not necessarily involve patients in their data collection with the high severity of injury the patient in question incurred. Through the literature review, few research subjects incurred the high degree of trauma and multiple fractures as the patient in this case report suffered. The current data is simply lacking in the medical community in any substantial amount. Thus, such considerations must be taken into account in the proper planning and management of a PVMVA polytrauma patient. Vascular injury could cause significant mortality, even though the patient may present with well-defined head trauma, and the national averages strongly suggest that focus be placed on the craniocerebral injuries.

The patient in question without a doubt is a victim of severe pelvic ring disruption and polytrauma. She had no rectal tone, which could be attributed to nerve injury. There was guaiac positive stool findings, which could indicate visceral perforation. Inspection of the Foley catheter revealed draining bright red blood, which could have been the result of urethral and/or bladder involvement. There was traumatic damage to vascular structures within the pelvis, leading to emergent arterial embolization. As stated once before, there is a dispute as to the proper protocol to treat patients with massive trauma. Treatment protocol is not as widely accepted due to difficulty in evaluation of the patient, varying degrees of severity of multiple injuries, the ability to gauge the life-threatening status of numerous injuries, institutional capabilities, healthcare provider experience, and so forth. Although this patient unfortunately passed away from her injuries, she and other in similar circumstances could benefit from a treatment protocol consensus among healthcare providers.

## 4. Conclusion

This case presentation glaring reveals that the patient incurred which is referred to as the “fatal triad,” which is under dispute in the orthopedic community. The patient displayed injury patterns which are in contrast to the commonly circulated thoughts of biodynamic mechanisms of fracture. This calls for more research in this field. Although she suffered from this triad, the patient did not succumb to what is widely accepted as the most deadly component, the head injury. This is despite the fact that she did suffer significant trauma in this region. She did suffer substantial spinal injury, not particularly common in comparison to other injuries in the PVMVA patient. The multiple visceral, vascular, and neurological injuries sustained by this patient are not uncommon in pelvic ring fractures, but the treatment protocol of such patients does not have a common consensus. The fact that the patient defied current trends of thought in the trauma community, along with lack of concrete consensus as to the treatment of severe trauma as in this case, all points to the need for further research into the field of PVMVA patients. This patient is a unique in that she may be considered an extreme trauma case. In the review of the literature, few subjects were found to have the high degree of trauma the patient in question incurred. In this regard, research is scarce due to the smaller number of severe trauma cases, and the high degree of associated mortality of such patients leads to a void in the data regarding widely accepted treatment protocols. More research in this arena is warranted. This lack of information contributes to the morbidity and mortality associated with such devastating injuries.

Overall it should be noted that the overlying theme displayed in the literature and data analyzed in this paper demonstrate the vital importance of the orthopedic surgeon in the management of the pedestrian versus motor vehicle accident patient. No matter the particular mechanism of injury, occurrence, or agreed-upon treatment protocol, the role of the orthopedic physician is instrumental to the wellbeing of the PVMVA trauma patient.

## References

[1] DOT-HS-810-994, P. P. Traffic Safety Facts. http://www.nhtsa.gov/.

[2] Demetriades D, Murray J, Martin M (2004). Pedestrians injured by automobiles: relationship of age to injury type and severity. *Journal of the American College of Surgeons*.

[3] Ameratunga S, Hijar M, Norton R (2006). Road-traffic injuries: confronting disparities to address a global-health problem. *The Lancet*.

[4] Landy DC, Norton RA, Barkin JA, Henriques S, Owens P, Miki RA (2010). Upper extremity fractures in pedestrian versus motor vehicle accidents: an underappreciated concern. *The Iowa Orthopaedic Journal*.

[5] Farley HH (1965). The fatal triad—skull, pelvis, extremity fractures. *Minnesota Medicine*.

[6] Waddell JP, Drucker WR (1971). Occult injuries in pedestrian accidents. *Journal of Trauma*.

[7] Brainard BJ, Slauterbeck J, Benjamin JB (1992). Fracture patterns and mechanisms in pedestrian motor-vehicle trauma: the ipsilateral dyad. *Journal of Orthopaedic Trauma*.

[8] Kong LB, Lekawa M, Navarro RA (1996). Pedestrian-motor vehicle trauma: an analysis of injury profiles by age. *Journal of the American College of Surgeons*.

[9] Markogiannakis H, Sanidas E, Messaris E (2006). Motor vehicle trauma: analysis of injury profiles by road-user category. *Emergency Medicine Journal*.

[10] Hill DA, Delaney LM, Duflou J (1996). A population-based study of outcome after injury to car occupants and to pedestrians. *Journal of Trauma*.

[11] Frakes MA, Evans T (2004). Major pelvic fractures. *Critical Care Nurse*.

[12] Wolinsky PR (1997). Assessment and management of pelvic fracture in the hemodynamically unstable patient. *Orthopedic Clinics of North America*.

[13] Coppola PT, Coppola M (2000). Emergency department evaluation and treatment of pelvic fractures. *Emergency Medicine Clinics of North America*.

[14] Egol KA, Koval KJ, Zuckerman JD (2010). *Handbook of Fractures*.

